# A Rare Presentation of Urethral Duplication in Conjunction With Anorectal Malformation Observed in a Male Infant

**DOI:** 10.1155/crpe/4725606

**Published:** 2025-04-23

**Authors:** Dinesh V. Hinge, Rajendra Saoji, Kiran Khedekar, Amar Taksande

**Affiliations:** ^1^Department of Pediatrics, Jawaharlal Nehru Medical College and Datta Meghe Institute of Higher Education and Research (Deemed to be University), Wardha 442001, Maharashtra, India; ^2^Department of Children's Surgical and Endoscopy Center, Nagpur 440010, Maharashtra, India; ^3^Department of Pediatrics Surgery, All India Institute of Medical Sciences, Nagpur 441108, Maharashtra, India

**Keywords:** anorectal malformation, colostomy, urethral duplication, urethroplasty, urogenital malformation

## Abstract

Anorectal malformation (ARM) refers to a group of congenital anomalies that affect the anus, rectum, and sometimes the urinary and reproductive tracts. A full-term male newborn was diagnosed with ARM and rectoperineal (scrotal) fistula during a first clinical screening examination at birth. He also had urethral duplication on the micturating cystourethrogram (MCUG) scan performed on Day 2 of life. The child underwent transverse colostomy at 24 h of life and corrective surgery (posterior sagittal anorectoplasty and urethroplasty) at 6 months of life, followed by colostomy closure after 3 months. This case highlights the importance of the first newborn clinical screening examination to rule out major congenital malformation and the thorough evaluation for associated urogenital defects in the case of ARM before definitive corrective surgeries for better clinical outcomes.

## 1. Introduction

Anorectal malformation (ARM) is a congenital condition where the anus and rectum do not develop properly. It often presents as an absent anal opening. The anus may be absent or improperly formed, leading to a blockage in the passwhere the anus and rectum do not develop properly. It often presents as an absent anal opening. The anus may be absent or improperly formed, leading to a blockage in the passage of stool. Over 95% of cases particularly males have a fistulous connection between the rectum and either the perineum or the urinary tract. ARM often present as an isolated malformation or as a part of VACTERL deformity. Common congenital malformations associated with ARM are 73% and urogenital malformations are present in more than > 32% of patients with ARM, as both entities develop from urogenital sinus [1]. However, urethral duplication is a rare occurrence that may be complete or incomplete. The exact etiology of this malformation is not entirely understood but is believed to be due to disruptions in the early stages of embryonic development. Urethral duplication can be classified as per Effmann's classification into three varieties [2]. Type I duplication is the most prevalent type and includes duplicated urethra which opens at the penis. It typically remains asymptomatic and does not need any intervention. Type II malformation is further classified into two subgroups: Type IIA and IIB. Type IIA is complete and patent duplication with two urethral meatuses, whereas Type IIB is patent duplication with a single urethral meatus. Type IIA can be further classified as IIAl, where noncommunicating urethras emerge independently from the bladder, and IIA2, where a second channel originates from the first; this variety is called as Y-type urethral duplication. Complete or partial caudal duplication is referred to as Type III duplication as shown in Table 1 [3].

ARMs are often missed on antenatal scans. This may be due to stringent laws regarding antenatal sex determination in countries such as India. First, a thorough clinical screening examination after initial stabilization of the newborn is critical to diagnose major congenital malformations such as ARM, VACTERL deformity, cleft palate, and disorders of sexual development (DSD).

A thorough diagnostic workup including micturating cystourethrogram (MCUG) should be performed to identify associated urogenital anomalies before performing corrective anorectal surgeries, as undiagnosed urethral malformations such as Y-type urethral duplication may lead to urethral damage during anoplasty.

## 2. Case Report

**Figure 1:**
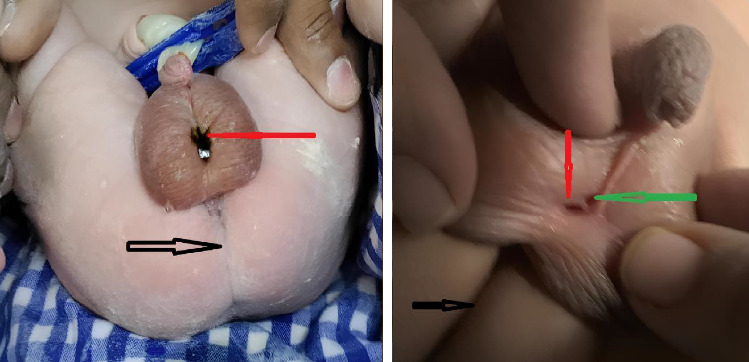
The well-defined abnormal opening in the midline raphe through which the baby passed a few drops of meconium (red arrow), urine from another opening (green arrow), and an absent anal opening (black arrow).

A full-term male newborn with a birth weight of 3.2 kg was delivered via lower segment caesarian section in view of the nonprogress of labor with failed induction. There was no significant antenatal history or any risk factors such as drug intake, radiation exposure, or alcohol intake during pregnancy. Antenatal anomaly scan (20 weeks of GA) and third-trimester scan last performed at 30 weeks of gestation were reported normal. There was no significant family history. Baby has an uneventful postnatal transition with normal vitals without any evidence of respiratory distress. On clinical examination at birth, he was noted to have an absent anal opening with a well-defined fistulous opening in the midline through which the baby passed a few drops of meconium at birth, as shown in Figure 1.

**Figure 2:**
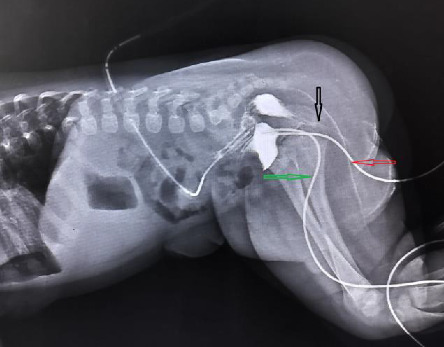
In a cross-table lateral decubitous film of distal loopogram, the green arrow shows the dorsal urethra, the red arrow shows the duplicated ventral urethra, and the black arrow shows the anorectum.

Both testes were palpable in the scrotum. He did not have any dysmorphic facial features, cleft lip, cleft palate, or any spine or limb defects. His external urethral opening was observed at the tip of the glans penis and there were two palpable testes. The abdomen was soft, with no organomegaly, and the central nervous and cardiorespiratory systems were within standard limits. After providing routine care in the delivery room, the baby was shifted to the NICU and put on complete intravenous fluid. Renal and liver function was normal, 2-D echocardiogram normal, and abdominal ultrasound was normal. A distal loopogram was performed and diagnosed as low ARM. A distal loopogram was performed in which contrast was pushed from the urethra and fistulous tract, and it was found that both were connected with the urinary bladder, suggesting urethral duplication in Figure 2.

**Figure 3:**
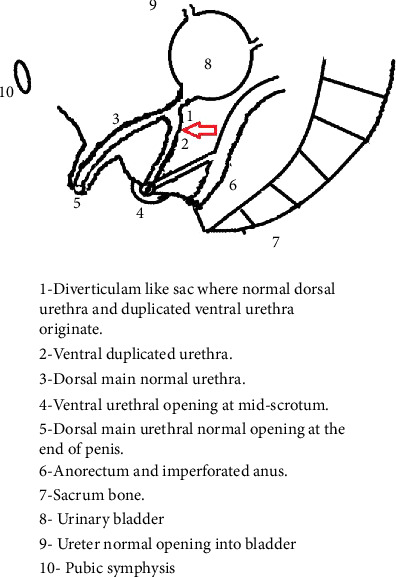
In the schematic representation, the duplication of the urethra is shown by the red arrow.

After admission to NICU, a routine hematological investigation was performed, and complete cell count, renal function, and liver function with coagulation were normal. On the second day of life, end colostomy was performed. After the MCUG, an examination under anesthesia (E.U.A.) and cystoscopy were performed. According to Effmann's classification, our patient was assigned to the Type IIA2 category as depicted in Figure 3.

**Figure 4:**
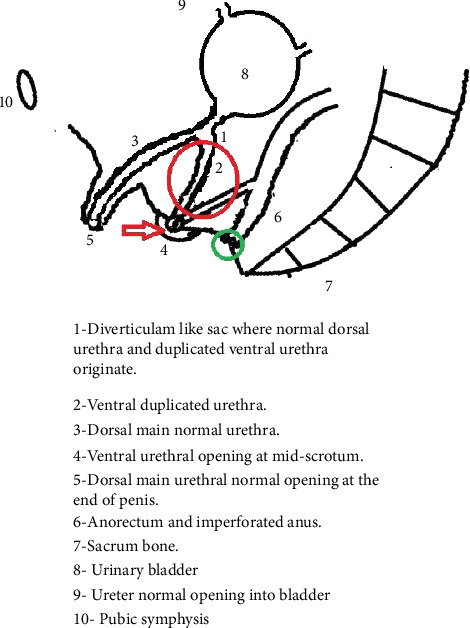
In the schematic representation, the red circle part was excised; in the green circle, area neo-anus was formed by PSARP; and the red arrow area was closed, which was the ureteral duplication site.

A guidewire was inserted via the midscrotal space, and urethroscopy revealed that it had entered the midbulbar urethra. There was a massive diverticulum in the sagittal plane of the proximal bulb. It included a small aperture through which the scope could enter the large cavity of the diverticulum. The diverticulum's mouth was enlarged. At four and a half months later, the procedure was used again. There was a significant decrease in the diverticulum in the third setting. A vertical scrotal incision was made in the third sitting to transfer the midscrotal duplication to the corpus spongiosum. For 2 weeks, the catheter remained in situ. Posterior sagittal anorectoplasty (PSARP) and urethroplasty have been accomplished as shown in Figure 4.

A closure colostomy was performed 2 months later. Parents suggested manual dilatation of the rectum using a rubber tube catheter for 3 months. After catheter removal, the child passed urine from the penile urethra and stool from the created anal opening. The child is followed up in pediatric O.P.D. and is doing well, passing urine from the penile urethra and stool from the rectum adequately. No urinary or stool incontinence was observed, and weight and height are normal for age. Neurodevelopment of a child is appropriate for age.

## 3. Discussion

Urethral duplication is a rare congenital anomaly and occurs more commonly in male infants. Urethral duplication along with ARM is even a rarer occurrence and less than 200 cases have been reported in the literature so far. Aristotle and Vesalius first described urethral duplication [3]. Haleblian et al. have reported 10 cases of “Y” urethral duplication, and seven of them had associated ARM; of which, three had high anomalies, one had anteriorly placed anus, and the rest three had anorectal stenosis [4]. The genesis and progression of Y-type urethral duplications have been the subject of many speculations and several ideas were put forward to explain the occurrence of the syndromic presentation. The underlying etiology is most likely heterogeneous and multifaceted in nature and comprises ischemia, aberrant Müllerian duct termination, and urogenital sinus development failure [5]. In contrast to other urethral duplication forms, Y-type duplication is often characterized by the association with other severe congenital malformations such as ARM. It can occur in isolation or as a part of VACTERL association (vertebral defects; anal atresia; cardiac defects; tracheoesophageal abnormalities including atresia, stenosis, and fistula; renal and radial anomalies; and limb defects) [6].

Treatment for ARM and urethral duplication requires early identification and thorough assessment of the patient as a whole. Patients should be assessed for the type of ARM and associated urogenital malformation before embarking on definitive repair. The surgical treatment primarily depends on the level of the rectal pouch. For the intermediated and high-type ARM, three staged procedures were performed which consist of colostomy during the neonatal period, radical repair at a later stage, and finally, the generally performed colostomy closure. While the majority of pediatric surgeons endorse this approach, some support a single-stage pull-through procedure during the newborn period, claiming that it provides superior postoperative bowel function [7]. The aim of the surgical therapy for urethral duplication poses a substantial challenge and should be established with good urethral reconstruction without any strictures or fistulae while maintaining continence. This requires multiple surgeries with varying results. Singal et al. reported two cases, where the intervening septum was excised endoscopically in one case, and in the other case, the duplicated urethra was removed [8]. Mane et al. reported a series of eight infants with urethral duplication and ARM. Among them, four patients were managed with single-stage repair, while the other three had multistage surgeries [9]. Coleman et al. reported two cases that needed different surgical approaches in order to retain continence and preserve the ventral urethra based on their anatomical and functional features [10]. Alladi et al. have also reported four urethral duplication cases with age of presentation ranging from a newborn to 10 years old and were treated by completely excising the auxiliary urethra and correcting related abnormalities [11]. Shah et al. reported the case of a 16-month-old male with duplication of urethra and diverticulum that was managed endoscopically [12]. Thapa et al. reported a case of a 24-year-old male that presented with discharge from the proximal part of the penis since birth and was diagnosed to have prepubic sinus and was managed by complete excision of the sinus tract [13].

## 4. Conclusion

This case reinforces meticulous newborn clinical examination by a pediatrician at birth in the delivery room for major congenital malformation for timely intervention and better outcomes. The approach to clinical management must be tailored to the specific congenital malformation, associated anomalies, and available resources. Surgical intervention is often required to reconstruct and repair the affected structures to restore normal function and achieve urinary and bowel continence. A multidisciplinary team including pediatricians, pediatric surgeons, and urologists is usually vital for managing this intricate ailment.

## Figures and Tables

**Table 1:** Effmann's classification of duplication of urethra.

Type	Description	Photographic representation
Type I	Duplicated urethra which opens on the penis	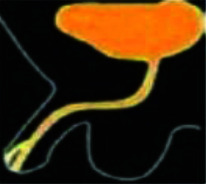

Type IIA1	Two independent urethras emerging from the bladder and opening with two meatuses	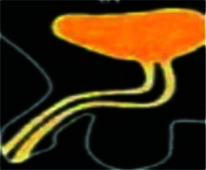

Type IIA2	Second channel arising from the first independent courses into a second meatus	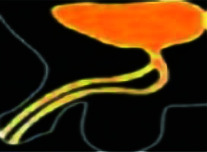

Type IIA2 (Y-type)	Prostatic urethra splits into two channels with one extending to the glans and the more functional ventral one coursing to the perineum near the rectum	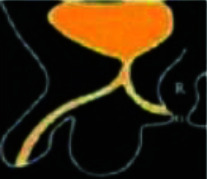

Type IIB	One meatus and two urethras arising from the bladder or posterior urethra unite in one channel	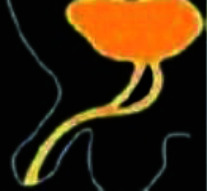

Type III	Urethral duplication as a component of partial or complete caudal duplication	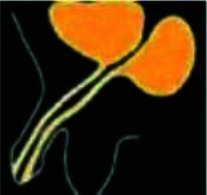

## Data Availability

The data supporting the conclusions of this report are contained within the report. Additional patient data are protected under patient privacy regulations and policies and will be provided on request.
